# Dynamic contrast-enhanced mammography and breast MRI in the diagnosis of breast cancer and detection of tumor size

**DOI:** 10.55730/1300-0144.5786

**Published:** 2023-12-11

**Authors:** Muhammed TEKİNHATUN, Nuran SABİR, Ergun ERDEM, Sevda YILMAZ, Furkan UFUK

**Affiliations:** 1 Department of Radiology, Faculty of Medicine, Dicle University, Diyarbakır, Turkiye; 2 Department of Radiology, Faculty of Medicine, Pamukkale University, Denizli, Turkiye; 3 Department of General Surgery, Faculty of Medicine, Pamukkale University, Denizli, Turkiye

**Keywords:** Breast cancer, dynamic breast magnetic resonance imaging, contrast-enhanced spectral mammography

## Abstract

**Background/aim:**

The aim of this study is to evaluate the performance of contrast-enhanced mammography (CEM) and dynamic breast MRI techniques for diagnosing breast lesions, assess the diagnostic accuracy of CEM’s using histopathological findings, and compare lesion size measurements obtained from both methods with pathological size.

**Materials and methods:**

This prospective study included 120 lesions, of which 70 were malignant, in 104 patients who underwent CEM and MRI within a week. Two radiologists independently evaluated the MR and CEM images in separate sessions, using the BI-RADS classification system. Additionally, the maximum sizes of lesion were measured. Diagnostic accuracy parameters and the receiver operating characteristics (ROC) curves were constructed for the two modalities. The correlation between the maximum diameter of breast lesions observed in MRI, CEM, and pathology was analyzed.

**Results:**

The overall diagnostic values for MRI were as follows: sensitivity 97.1%, specificity 60%, positive predictive value (PPV) 77.3%, negative predictive value (NPV) 93.8%, and accuracy 81.7%. Correspondingly, for CEM, the sensitivity, accuracy, specificity, PPV, and NPV were 97.14%, 81.67%, 60%, 77.27%, and 93.75%, respectively. The ROC analysis of CEM revealed an area under the curve (AUC) of 0.907 for observer 1 and 0.857 for observer 2, whereas MRI exhibited an AUC of 0.910 for observer 1 and 0.914 for observer 2. Notably, CEM showed the highest correlation with pathological lesion size (r = 0.660 for observer 1 and r = 0.693 for observer 2, p < 0.001 for both).

**Conclusion:**

CEM can be used with high sensitivity and similar diagnostic performance comparable to MRI for diagnosing breast cancer. CEM proves to be a successful diagnostic method for precisely determining tumor size.

## 1. Introduction

Breast cancer is the most prevalent malignancy affecting women, and mammography serves as the primary screening tool for early diagnosis, thereby enhancing the life expectancy of women at risk [1–3]. While conventional mammography exhibits a relatively high sensitivity for lesion detection, this sensitivity and specificity are significantly reduced in women with dense breast tissue [4,5]. In contrast, contrast-enhanced magnetic resonance imaging (MRI) is an effective method for identifying and characterizing breast lesions, particularly in women with dense breast tissue, thereby reducing unnecessary biopsies [4,6]. However, MRI requires 25–30 min, and the vast number of sequences obtained in MRI significantly prolongs the time required for interpretation. Moreover, limited access to MRI, its expensive and time-consuming nature, and contraindications for patients with metallic implants, renal failure, and pacemakers are limitations of breast MRI [4,6,7].

Recent research highlights the potential of contrast-enhanced mammography (CEM) as a promising tool for identifying breast lesions based on contrast enhancement patterns and intensity relative to breast glandular tissue [2,8]. CEM requires only 10 min and produces four easily interpretable images. CEM has shown significant improvements in sensitivity and specificity for breast cancer diagnosis compared to conventional mammography, displaying performance comparable to that of breast MRI [2,8–10]. CEM offers advantages such as reduced examination time, more straightforward implementation, and a significantly lower cost than MRI. However, there is a limited number of prospective studies assessing the diagnostic efficiency of CEM in breast lesions compared to histopathology.

The size and extent of index breast tumor are essential factors for accurate staging. Several studies have shown that the tumor sizes measured on CEM images closely correspond to the actual tumor sizes measured during surgery [11–13]. However, limited studies have evaluated lesion size on MRI and CEM, and there is insufficient data. In this prospective study, we aim to compare the diagnostic efficiency of CEM and dynamic breast MRI methods for diagnosing breast lesions and to evaluate the diagnostic performance of CEM based on histopathological results. We also aim to measure the lesion size in CEM and MRI and compare it with the pathological size.

## 2. Patients and methods

### 2.1. Study population

This prospective study was approved by the local clinical research ethics committee (approval number 60116787-020/84836 from the “Pamukkale University Non-Interventional Clinical Research Ethics Committee”), and informed consent was obtained from all participating patients.

Consecutive patients with contrast-enhanced breast MRI indications between January 2016 and May 2018 were assessed. Patients with metal implants, a known sensitivity to contrast agent, pregnancy, lactation, renal failure, or systemic conditions such as hyperthyroidism and claustrophobia were excluded from the study. A total of 154 patients with MRI indications (problem-solving [n = 80], staging [n = 14], and screening high-risk women [n = 60]), without contraindications and who agreed to participate in the study, were prospectively analyzed. These 154 patients underwent both contrast-enhanced breast MRI and CEM within a week.

### 2.2. Contrast-enhanced mammography (CEM) protocol

All CEM examinations in our clinic were conducted using a digital mammography device (Selenia Dimensions; Hologic) with a dual-energy protocol. Subsequently, a 1.5 mL/kg (max. 100 mL) intravenous dose of a nonionic, water-soluble contrast agent (Omnipaque; 350 mg/mL, GE Healthcare) was administered via an automatic injector at a rate of 2 mL/s, without breast compression. Approximately 2 min postinjection, both breasts were positioned for CEM imaging, similar to conventional mammograms. Each CEM examination involved the acquisition a pair of low-energy and high-energy images. Four routine views were obtained within 5 min, starting 2 min after injection. Both breasts were imaged using a single dose of contrast agent, resulting in four images in two standard positions. The image acquisition sequence included cranio-caudal (CC) and mediolateral oblique (MLO) images for each breast. After postprocessing, I-view software (Hologic, Bedford, USA) was used to obtain subtraction images from both low- and high-energy acquisitions, highlighting the enhancement of the injected iodine.

### 2.3. Magnetic resonance imaging (MRI) protocol

All MRI examinations were conducted using an eight-channel breast coil on a 1.5-T MRI system (Signa HDx, GE Medical System), with patients positioned in the prone position. Intravenous injections of gadoterate meglumine (Dotarem, Guerbet) at 0.2 mmol/kg were administered as a single intravenous bolus using an automated syringe at a 2 mL/s rate, followed by a 20 mL saline flush. For the dynamic sequence, nine consecutive scans were obtained before and after the contrast agent injection, each with a single scan time of 60 s and comprising 64 single-phase scan slices. The standard MRI protocol includes axial 2-D T2W short tau inversion recovery (STIR, repetition time [TR]; 7475 ms, echo time [TE]; 54 ms, slice thickness; 3 mm, interslice gap; 1.5 mm), axial T2W TSE (TR; 5200 ms, TE; 85 ms, slice thickness; 3 mm, interslice gap; 0.5 mm), axial DWI (TR; 3000 ms, TE; 52 ms, slice thickness; 3 mm, interslice gap; 1.5 mm), axial precontrast T1W (TR; 600 ms, TE; 10 ms, slice thickness; 3 mm, interslice gap; 1.5 mm), and postcontrast dynamic axial T1W without fat suppression following intravenous contrast administration (TR; 6.1 ms, TE; 3 ms, slice thickness; 2.8 mm, no interslice gap). Subtraction and maximum intensity projection (MIP) images were also generated.

### 2.4. Image analysis

Breast MRI and CEM images were independently evaluated by two radiologists (observer 1, 23 years of experience in breast imaging; observer 2, two years of experience in breast imaging) in separate sessions, with at least a one-month interval between the evaluations. Observers were blinded to the histopathological results of the lesions, patient’s clinical information, and other imaging findings. Two observers independently measured the lesion sizes along the longest axis in the contrast-enhanced MR and CEM images. The specimen lesion size was measured by a pathologist in patients who underwent a mastectomy, and the pathological lesion size was used for reference in the study. Observer 1 evaluated and measured mammography and MRI separately during the reporting and study phases. The second observer performed evaluations only during the study phase.

The findings from MRI and CEM were evaluated according to the last edition of the Breast Imaging Reporting and Data System (BI-RADS) data dictionary, revised in 2013 [14]. The CEM examination produced both low-energy and recombined subtraction images. Low-energy images were initially interpreted to evaluate morphological abnormalities, and subtraction images were subsequently used to support the interpretation by focusing on contrast-enhancing areas. Although a dedicated BI-RADS classification for CEM is not yet available, our study used mammographic lexicons for low-energy images and MRI lexicons for contrast images, as recommended until a specific dictionary is established. Visual assessments were conducted based on the degree of contrast, and images were categorized as no enhancement, minimal, moderate, or marked contrast enhancement [15]. Nonmass-like enhancement lesions were also evaluated based on the BI-RADS criteria for both MRI and CEM, and they were classified as focal, linear segmental, regional, or multiple regional. All asymmetric appearances without contrast enhancement in CEM were classified as focal asymmetry. Segmental nonmass enhancement and microcalcifications in low-dose images are considered indicative of malignancy.

### 2.5. Statistical analysis

The data were analyzed using MedCalc and SPSS v25.0 software. The chi-square analysis was used to examine differences between categorical variables. Receiver operating characteristic curve (ROC) analysis assessed diagnostic performances. The Wilcoxon paired two samples test was employed to examine differences, and Pearson correlation coefficients (r) were used to quantify the strength and direction of linear relationships between continuous variables. Weighted kappa coefficients (κ) and Bland-Altman graphs were used to assess interobserver agreement. Kappa (κ) value indicating agreement were categorized as follows: κ = 0.20 for insignificant; κ = 0.21–0.40 for minimal; κ = 0.41–0.60 for moderate; κ = 0.61–0.80 for substantial; κ = 0.81–1.00 for excellent agreement. A p-value < 0.05 was considered statistically significant.

## 3. Results

**Figure 1:**
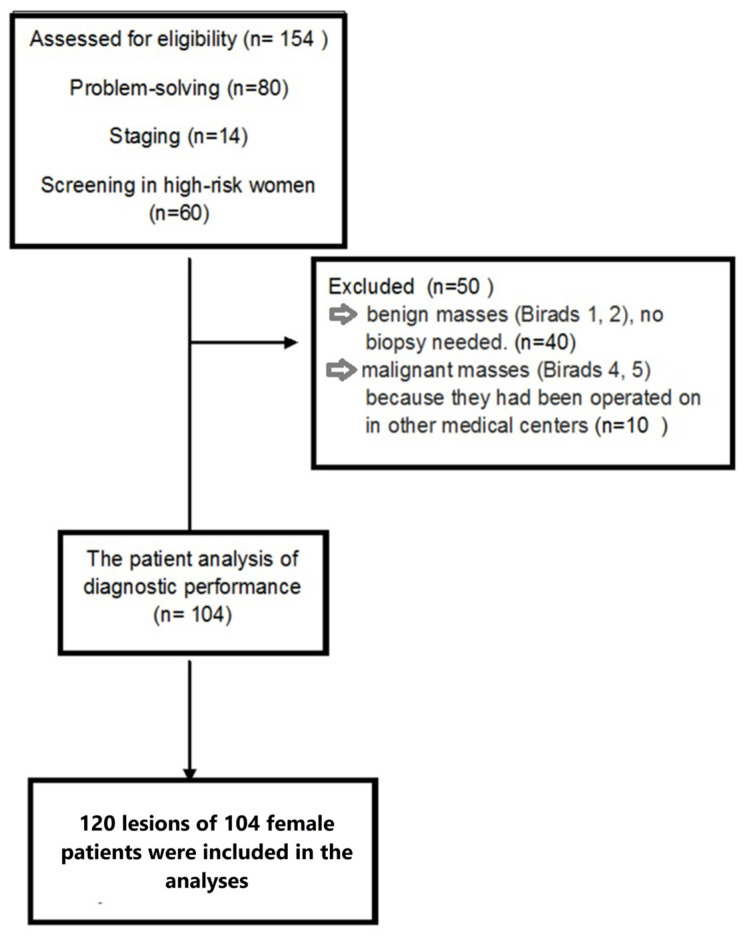
Flow diagram of the study population.

Fifty of 154 patients (32.5%) were excluded from the analyses due to the lack of histopathological results (Figure 1). A total of 120 lesions of 104 female patients, which were assessed histopathologically, were included in the analyses. Seventy lesions (58.3%) were malignant, and 50 lesions (41.7%) were benign, histopathologically. Thirty-nine patients underwent a mastectomy. The most common malignant lesions were invasive ductal carcinoma (IDC; 77.1%), while the fibrocystic disease was the most prevalent benign lesion (36%) (Table 1).

The average age of patients was 47.6 ± 10.1 years. Patients with malignant lesions were significantly older than those with benign lesions (45.1 ± 7.8 vs. 48.7 ± 10.7, p = 0.037).

### 3.1. CEM findings

**Figure 2:**
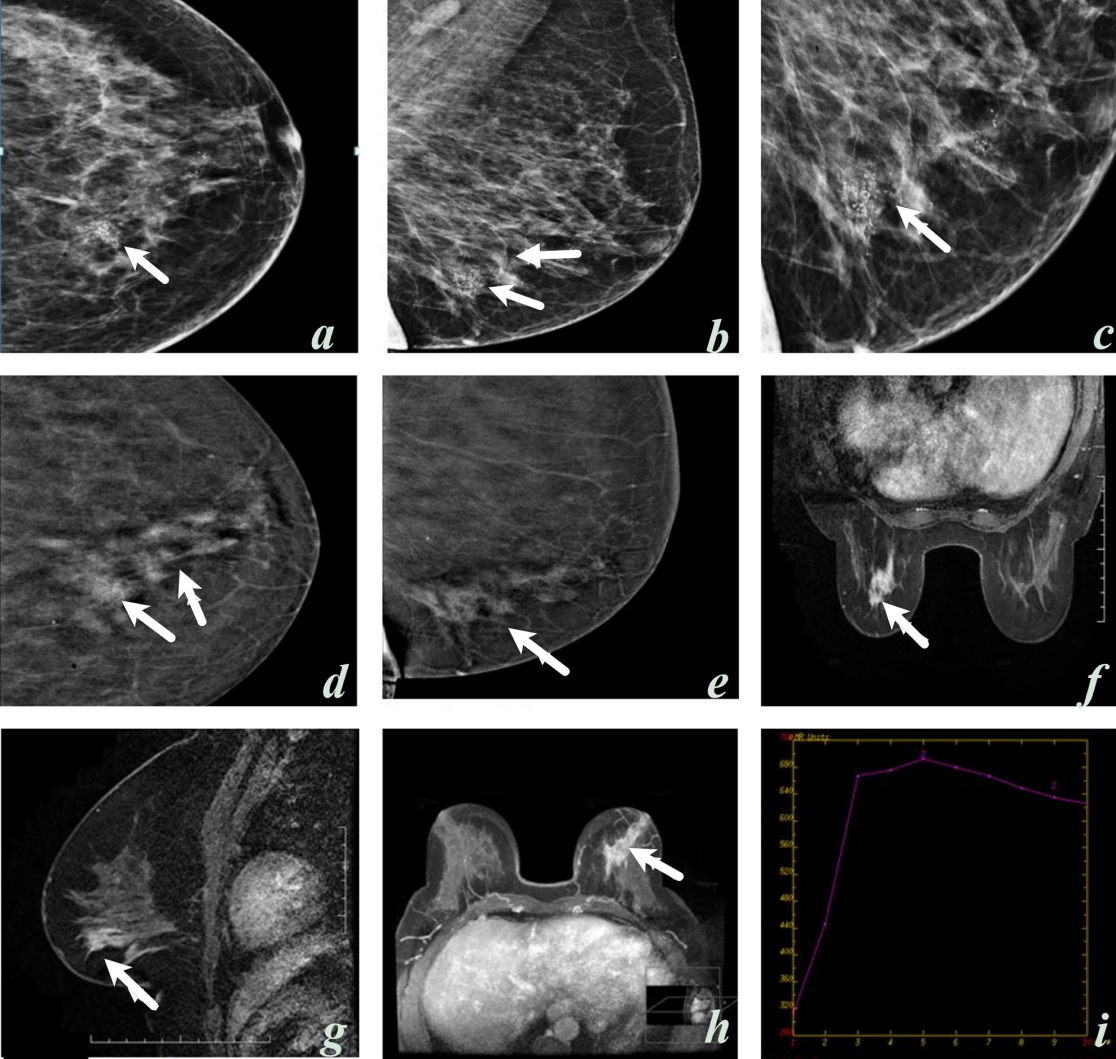
A 58-year-old woman, craniocaudal (**a, c**) and mediolateral oblique (**b**) low-energy contrast-enhanced mammography (CEM) images; suspicious microcalcifications with a thin pleomorphic and linear branching morphology with segmental distribution in the left inner lower quadrant (arrows). Contrast-enhanced mammography with subtraction (**d, e**), MRI dynamic (**f**), contrast sagittal (**g**), and three-dimensional (**h**) images display nonsegmental contrast enhancement (arrows). A type 3 (early enhancement and washout in delayed phase images) contrast enhancement pattern (**i**) was also observed in this area. Observer 1: BI-RADS 5 in CEM, BI-RADS 4 in MRI. Observer 2: BI-RADS 5 in CEM and MRI. The histopathological diagnosis of the lesion was invasive ductal carcinoma.

Twenty lesions (16.7%) showed no contrast enhancement in CEM, while 100 lesions showed contrast enhancement. Two malignant lesions (3%) had no contrast enhancement, while 68 (97%) had contrast enhancement. Notably, the two lesions without contrast enhancement on CEM had suspicious microcalcifications indicative of malignancy, and the histopathological diagnoses were invasive ductal carcinoma (IDC) and in situ ductal carcinoma (Figures 2a–2i). Contrast enhancement was not observed in 36% (n = 18) of benign lesions, while minimal contrast enhancement was observed in 32% (n = 16). Of the 81 lesions with moderate and marked enhancement, 65 (80.2%) were diagnosed as malignant and 16 (19.8%) as benign (Tables 2–4). Observer 1’s evaluation indicated that intense enhancement in CEM had a high predictive value for malignancy (AUC of 0.850, 95% CI; 0.773–0.926).

**Figure 3:**
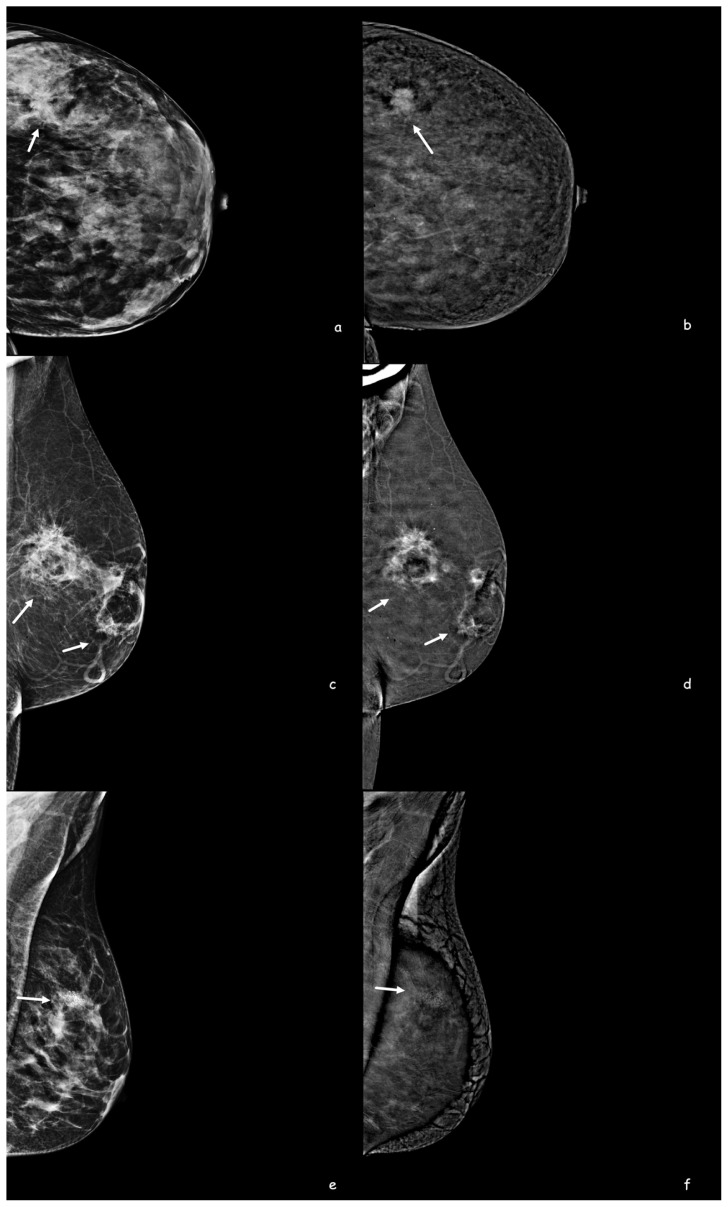
A 34-year-old woman, craniocaudal (**a**,) low-energy contrast-enhanced mammography (CEM) images, in the lower outer quadrant of the left breast (arrows), which is difficult to choose due to the dense breast pattern, with high-density, irregularly shaped margins that cannot be clearly seen, intense contrast enhancement in CEM subtraction (**b**) images. An irregularly shaped spiculated contoured mass lesion is observed (arrows). The diagnosis was invasive ductal carcinoma. A 63-year-old woman, a high-density mass in the left breast retroareolar and upper outer quadrant in MLO (**c**) low-energy CEM images (arrows), and an irregularly shaped spiculated contoured central fat-density mass with intense contrast enhancement on CEM subtraction (**d**) images lesions are observed (arrows). The diagnosis was fat necrosis. A 40-year-old woman has clustered suspicious microcalcifications in the left upper outer quadrant in MLO (**e**) low-energy CEM images, and there is no contrast enhancement at this level in CEM subtraction (**f**) images. At this level, the artifact of microcalcifications is seen. The lesion was misdiagnosed as MRI did not show contrast, and the diagnosis was ductal carcinoma in situ.

**Figure 4:**
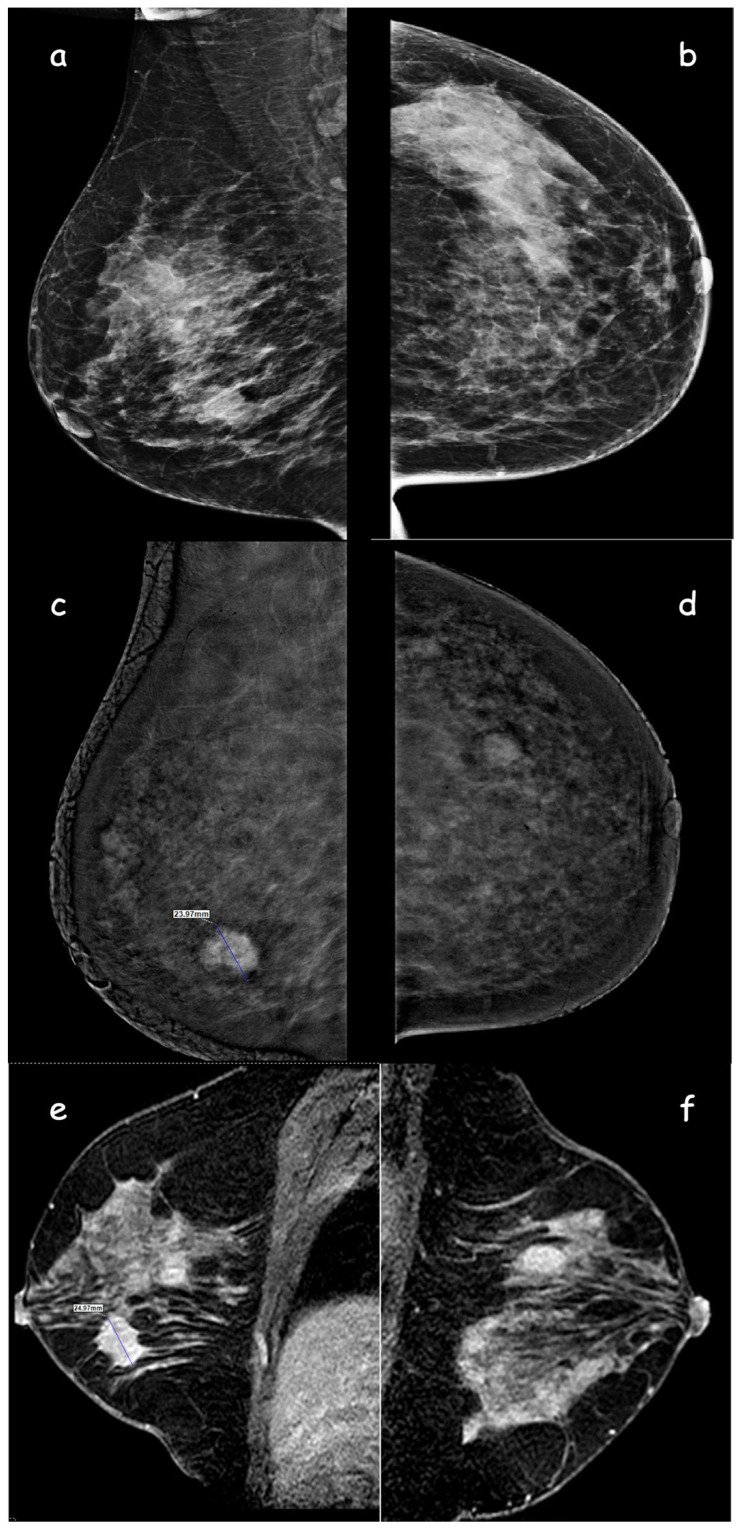
A 44-year-old woman, lesions could not be distinguished in MLO (**a**) and CC (**b**) low-energy CEM images due to the heterogeneous dense breast pattern. Subtraction images show an irregularly circumscribed lesion with intense contrast enhancement in the right breast lower outer quadrant (**c**) and another lesion with well-circumscribed minimal contrast enhancement in the left breast lower outer quadrant (**d**). On MRI, sagittal contrast-enhanced sections (**e, f**) show an irregular mass lesion on the right and a well-defined mass lesion on the left. The histopathological diagnosis of the lesion in the left breast was fibroadenoma, and the lesion in the right breast was invasive ductal carcinoma. Lesion size measurement along the longest axis on contrast enhanced CEM (**c**) and MRI images (**e**).

Malignant lesions predominantly exhibited spiculated margins (70.9%) as depicted in Figures 3a and 3b, while circumscribed contours were the least prevalent (1.8%). Benign lesions most commonly exhibited circumscribed contours (60.9%), and while irregular margins were the least common (17.4%) (Figures 3c and 3d). The positive predictive value of spiculated and irregular borders on CEM was 85.7% (Figures 3e and 3f). In terms of benignity, the positive predictive value of the circumscribed contour on CEM was 93%. While 27.3% of malignant mass lesions were irregular, 17.4% of benign lesions had an irregular shape. Observer 1’s evaluation showed that breast density in CEM had a high predictive value for malignancy (AUC of 0.707, 95% CI; 0.612–0.802) (Figures 4a–4d).

### 3.2. MRI findings

Malignant lesions most commonly exhibited spiculated margins (68.9%) and least commonly displayed smooth margins (1.6%) on MRI. In contrast, benign masses were most commonly characterized by smooth margins (76.9%) and least commonly by spiculated margins (2.6%). The positive predictive value for malignancy, when considering spiculated and irregular border structures, was found to be 87%, with an AUC of 0.860 (95% CI; 0.790–0.930). Meanwhile, the positive predictive value for benignity with smooth contours was 96%. Furthermore, 88.5% of malignant mass lesions had irregular shapes, whereas only 17.9% of benign lesions exhibited irregular shapes.

In malignant masses, 26 (42.6%) exhibited homogeneous contrast enhancement, 34 (55.7%) displayed heterogeneous enhancement, and 1 (1.7%) showed circular enhancement on MRI. Concerning benign masses, 13 (33.3%) exhibited homogeneous enhancement, 8 (20.5%) showed heterogeneous enhancement, and 6 (15.4%) displayed circular enhancement. Notably, 12 (30.8%) benign lesions exhibited no detectable enhancement. Of the 42 heterogeneously enhanced lesions, 34 (81%) were diagnosed as malignant, and 8 (19%) were benign. Early contrast enhancement and washout in the delayed phase MR images was an essential finding for malignancy, with an AUC of 0.888 (95% CI, 0.824–0.952) (Figures 4e and 4f).

### 3.3. Diagnostic performances of CEM and MRI

When considering BI-RADS-1, BI-RADS-2, and BI-RADS-3 lesions as benign and BI-RADS-4 and BI-RADS-5 as malignant, the overall diagnostic values of MRI showed a sensitivity of 97.1%, specificity of 60%, PPV of 77.3%, NPV of 93.8%, and accuracy of 81.7%. For dense breasts (type C and type D), the diagnostic values of MRI were as follows: sensitivity 97.1%, specificity 58.5%, PPV 66.67%, NPV 96%, and accuracy 76.32%. In nondense breasts (type A and type B), the diagnostic values of MRI were as follows: sensitivity 97.14%, specificity 66.67%, PPV 91.89%, NPV 85.71%, and accuracy 90.91%.

The diagnostic values for CEM in dense breasts (type C and type D) were as follows: sensitivity 97.14%, specificity 58.54%, PPV 66.67%, NPV 96%, and accuracy 76.32%. In nondense breasts (type A and type B), the diagnostic values of CEM were as follows: sensitivity 97.14%, specificity 66.67%, PPV 91.89%, NPV 85.71%, and accuracy 90.91%.

**Figure 5:**
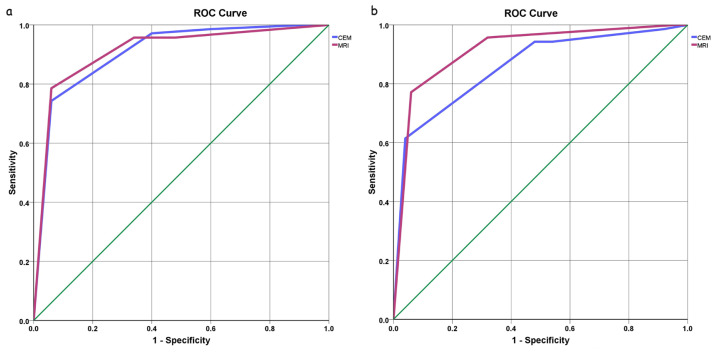
Receiver operating characteristic (ROC) curves for differentiating malignant breast masses from benign ones on CEM and MRI for **a) o**bserver 1 and **b)** observer 2.

With a cut-off value of BI-RADS > 3, the ROC analysis for CEM demonstrated an area under the curve (AUC) of 0.907 (95% confidence interval [CI], 0.852–0.963) for observer 1 (Figures 5a and 5b) and 0.857 (95% CI, 0.790–0.924) for observer 2. The ROC analysis for MRI demonstrated an AUC of 0.910 (95% CI, 0.854–0.967) for observer 1 and 0.914 (95% CI, 0.859–0.969) for observer 2 (Figures 5a and 5b).

### 3.4. Interobserver agreement

This study showed substantial agreement between the observers regarding the criteria used for BI-RADS scoring in CEM, with a kappa value (κ) of 0.638. Similarly, substantial agreement was observed among the observers for the criteria used for BI-RADS scoring in MRI, with a weighted kappa value (κ) of 0.739. Additionally, there was a substantial agreement between observer 1’s BI-RADS scores for both CEM and MRI (κ = 0.705) and between observer 2’s BI-RADS scores for CEM and MRI (κ = 0.649).

### 3.5. Intraobserver agreement

Observer 1 evaluated mammography and MRI separately during the reporting and study phases. The first observer’s intraobserver agreement kappa value for lesion size was 0.75 in CEM and 0.78 in MRI. Regarding the BI-RADS score, the intraobserver kappa value was 0.82 in CEM and 0.85 in MRI.

### 3.6. Lesion size

**Figure 6:**
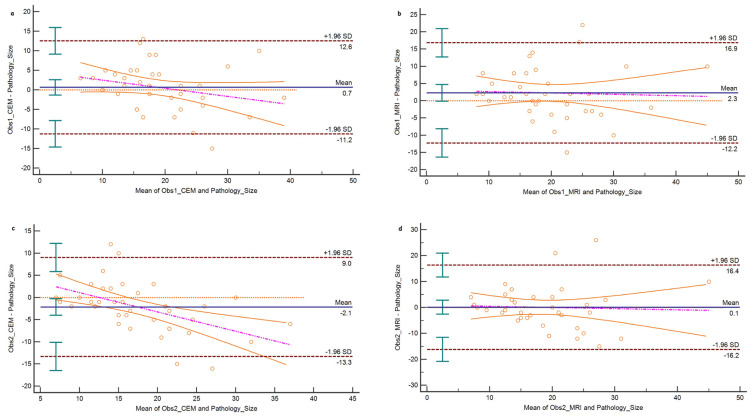
Bland-Altman plots for breast lesion size measurements compared to the pathological assessment. **a)** Observer 1 measurements on CEM, **b)** observer 1 measurement on MRI, **c)** observer 2 measurements on CEM, and **d)** observer 2 measurements on MRI.

Lesion sizes on CEM and MR images were evaluated in 39 patients who underwent a mastectomy. CEM showed the highest correlation with pathological lesion size, with r = 0.660 for observer 1 and r = 0.693 for observer 2 (p < 0.001 for both). MRI had a correlation of r = 0.588 for observer 1 and r = 0.546 for observer 2 (p < 0.001 for both). The Bland-Altman analysis of CEM lesion measurements revealed a bias of 0.7 mm (95% CI, −1.3 to 2.63) for observer 1 and −2.1 mm (95% CI, −3.98 to −0.28) for observer 2 (Figures 6a and 6b). In MR images, the Bland-Altman analysis showed a bias of 2.3 mm (95% CI, −0.1 to 4.71) for observer 1 and 0.1 mm (95% CI, −2.59 to 2.79) for observer 2 (Figures 6c and 6d).

## 4. Discussion

In this study, 120 histopathologically assessed breast lesions (58.3% were malignant and 41.7% were benign) were included in the analysis. Patients with malignant lesions were significantly older than those with benign lesions. Findings from contrast-enhanced mammography (CEM) indicated that 97% of malignant lesions had contrast enhancement, while 36% of benign lesions had no contrast enhancement. The positive predictive value of spiculated and irregular borders on CEM was 85.7%, whereas the positive predictive value for benignity with the circumscribed contour on CEM was 93%. Additionally, the positive predictive value for malignancy with spiculated and irregular border structures on MRI was 87%, while the positive predictive value for benignity with smooth contours was 96%. The diagnostic performances of CEM and MRI were similar, showing high sensitivity and specificity in detecting malignant lesions. A substantial interobserver agreement was observed for the criteria used for BI-RADS scoring in both CEM and MRI. Finally, CEM showed the highest correlation with pathological lesion size in patients who underwent a mastectomy.

Contrast-enhanced mammography (CEM) is an emerging tool similar to MRI that uses iodinated contrast medium to visualize breast neovascularity. Vessels formed through angiogenesis often leak the contrast medium, which then diffuses within tumor tissue, producing a contrast-enhanced image [2,8]. This enables the detection of malignant tumors despite dense breast tissue. In CEM, a dual-energy mammogram is obtained approximately 120 s after administering an iodinated contrast medium. CEM excels in displaying anatomical distortion and alterations in breast perfusion, likely due to tumor neovascularity [2,8,9,12,13,16]. A study by Cheung et al. [17] showed that CEM significantly increased sensitivity and specificity of digital mammography. Moreover, Fallenberg et al. [18] reported that 93% of cancers detected by CEM, but not by digital mammography, were found in women with dense breast tissue. The reported sensitivity and specificity for CEM in the literature are 93–100% and 63–88%, respectively, with NPV ranging from 92% to 100% and a higher PPV than breast MRI [8,12,13]. However, there are several factors to consider when using CEM to evaluate ambiguous imaging findings. Firstly, benign lesions can display enhancement, as shown in the present study. Secondly, cysts exhibit a distinct appearance in CEM. In low-energy images, cysts typically appear as well-defined, round, or oval masses, with no enhancement except for a thin enhancing rim. Similar to breast MRI, an inflamed cyst may present with a thick enhancing wall [8].

CEM may offer some benefits in assessing suspicious microcalcifications. In the present study, two lesions with suspicious microcalcifications indicative of malignancy were diagnosed with IDC and in situ ductal carcinoma. Therefore, suspicious calcifications on CEM should be biopsied regardless of enhancement. While Cheung et al. [19] reported that enhancement of microcalcifications suggested underlying malignancy, there was no enhancement in two cases in our study. Consistent with our findings, Houben et al. [20] reported no significant difference in diagnostic performance between CEM and conventional mammography when evaluating suspicious calcifications.

While contrast-enhanced breast MRI is recommended for women at a high risk of developing breast cancer, there is a substantial population of women at an intermediate risk [21,22]. CEM is well-tolerated by patients, and many express a preference for it over MRI due to its quicker, more comfortable, and less noisy nature. Moreover, the results of the present study demonstrated that CEM has high sensitivity and specificity in patients with dense breasts. Therefore, CEM can be considered a successful alternative diagnostic method for women who are either unable or unwilling to undergo MRI.

Jochelson et al. [23] prospectively evaluated 307 women using MRI and CEM. They reported that the specificity of CEM was equivalent to that of MRI [23]. Similarly, Sung et al. [24] showed that CEM had a cancer detection rate similar to that reported for MRI. A metaanalysis revealed that that the sensitivity of CEM approaches that of breast MRI and is likely more specific [25]. Consistently, the results of the present study showed that the diagnostic performances of CEM and MRI were similar, with both showing high sensitivity and specificity in detecting malignant lesions.

Several studies have demonstrated that breast lesion sizes measured using CEM ranged from 0.03 mm to 5 mm, as compared to the tumor sizes determined during surgery. Similarly, the present study showed that tumor sizes measured using CEM ranged from 0.7 mm to 2.1 mm, in comparison to the pathological tumor size [11–13]. Fallenberg et al. [18] showed that CEM had the highest correlation with surgical specimens (r = 0.73 for CEM and r = 0.65 for MRI, p < 0.001 for all). Similarly, in the present study, CEM showed the highest correlation with pathological lesion size, with r = 0.660 for observer 1 and r = 0.693 for observer 2 (p < 0.001 for both). However, Kim et al. [16] reported better accuracy for MRI than for CEM in terms of tumor size (r = 0.84 for MRI and r = 0.77 for CEM, p < 0.001 for all). The differences in tumor size measurement accuracy between studies could be attributed to various factors, such as the expertise of the observers, the quality and resolution of the contrast-enhanced images, and the specific types of breast cancer.

This study has some limitations. First, excluding lesions without a histopathological diagnosis resulted in a smaller number of benign lesions and a decrease in specificity value. Second, the study included 120 breast lesions. This may limit the generalizability of the findings to a broader population. Third, the study utilized two radiologists with different levels of experience in breast imaging (23 years and two years) to evaluate the breast MRI and CEM images. While the interobserver agreement was assessed, the difference in experience may still introduce variability in the diagnostic performance of the two imaging methods. Lastly, the study used mammographic lexicons for low-energy images and MRI lexicons for contrast images, following recommendations until a specific dictionary is established. This may affect the consistency and comparability of the findings with other studies.

In conclusion, CEM can be used with high sensitivity and comparable diagnostic performance to MRI for diagnosing breast cancer. CEM is an essential diagnostic method, demonstrating high sensitivity and NPV similar to MRI. It can serve as a valuable alternative to MRI for diagnosing breast lesions when necessary. However, further studies with larger patient samples and broader diagnostic subgroups are needed to clarify the diagnostic performance of CEM more comprehensively.

## Figures and Tables

**Table 1:** The histopathological diagnoses of breast lesions.

Benign lesions	n (%)	Malignant lesions	n (%)
**Fibrocystic disease**	18 (36)	Invasive ductal carcinoma	54 (77.1)
**Fibroadenoma**	11 (22)	Invasive ductal carcinoma with ductal carcinoma in situ	8 (11.4)
**Cyst**	5 (10)	Invasive lobular carcinoma	3 (4.3)
**Abscess**	3 (6)	Ductal carcinoma in situ	2 (2.9)
**Intraductal papilloma**	3 (6)	Medullary carcinoma	1 (1.4)
**Mastitis**	3 (6)	Metaplastic carcinoma	1 (1.4)
**Sclerosing adenosis**	2 (6)	Tubular carcinoma	1 (1.4)
**Granulomatous inflammation**	2 (4)		
**Atypical ductal hyperplasia**	1 (2)		
**Fat necrosis**	1 (2)		
**Apocrine metaplasia**	1 (2)		
**Total**	**50 (100)**		**70 (100)**

**Table 2:** The CEM and MRI characteristics of the benign and malignant lesions according to the observers’ evaluation.

	CEM				MRI			
	Malignant lesions n (%)		Benign lesions n (%)		Malignant lesions n (%)		Benign lesions n (%)	
	Obs 1	Obs 2	Obs 1	Obs 2	Obs 1	Obs 2	Obs 1	Obs 2
**Mass shape**								
**Oval/round**	10 (14.2)	12 (17.1)	23 (46)	27 (54)	7 (10)	13 (18.5)	32 (64)	31 (62)
**Irregular**	45 (64)	39 (55.7)	8 (16)	8 (16)	54 (77.1)	48 (68.6)	7 (14)	7 (14)
**Margins**								
**Circumscribed**	1 (1.4)	1 (1.4)	22 (44)	27 (54)	1 (1.4)	1 (1.4)	30 (60)	27 (54)
**Irregular**	15 (21.4)	15 (21.4)	4 (8)	6 (12)	18 (25.7)	15 (25.7)	8 (16)	6 (16)
**Spiculated**	39 (55.7)	45 (64.3)	5 (10)	5 (10)	42 (60)	45 (60)	1 (2)	5 (2)
**Enhancement pattern**					**Kinetic curve**			
**Absent (CEM)/No enhancement (MRI)**	2 (2.8)	3 (4.3)	18 (36)	18 (36)	2 (2.8)	3 (4.3)	12 (24)	10 (20)
**Minimal (CEM)/ persistent (type I) (MRI)**	3 (4.3)	3 (4.3)	16 (32)	10 (20)	3 (4.3)	4 (5.7)	23 (46)	27 (54)
**Moderate (CEM)/plateau (type II) (MRI)**	10 (14.2)	3 (4.3)	7 (14)	6 (12)	13 (18.5)	10 (14.3)	11 (22)	6 (12)
**Marked (CEM)/washout (type III) (MRI)**	55 (78.5)	61 (87.1)	9 (18)	16 (32)	52 (74.4)	53 (75.7)	4 (8)	7 (14)

CEM, contrast-enhanced mammography; MRI, magnetic resonance imaging; Obs, observer.

**Table 3:** The CEM and MRI characteristics of the benign and malignant lesions according to the observers’ evaluation.

	CEM				MRI			
	Malignant lesions n (%)		Benign lesions n (%)		Malignant lesions n (%)		Benign lesions n (%)	
	Obs 1	Obs 2	Obs 1	Obs 2	Obs 1	Obs 2	Obs 1	Obs 2
**Nonmass+mass enhancement**	3 (4.3)	0 (0)	0 (0)	0 (0)	0 (0)	0 (0)	0 (0)	0 (0)
**Nonmass enhancement**								
**Focal**	3 (4.3)	1 (1.4)	6 (12)	4 (8)	0 (0)	0 (0)	3 (6)	5 (10)
**Linear**	0 (0)	0 (0)	1 (2)	0 (0)	0 (0)	5 (7.1)	1 (2)	3 (6)
**Segmental**	10 (14.2)	12 (17.1)	1 (2)	3 (6)	6 (8)	0 (0)	3 (6)	1 (2)
**Regional**	2 (2.8)	3 (4.3)	1 (2)	0 (0)	1 (1.4)	0 (0)	2 (4)	0 (0)
**Multiple regional**	1 (1.4)	0 (0)	0 (0)	0 (0)	0 (0)	1 (1.4)	2 (4)	0 (0)
**Nonmass enhancement pattern**								
**Homogeneous**	1 (1.4)	0 (0)	0 (0)	0 (0)	2 (2.8)	0 (0)	5 (10)	1 (10)
**Heterogeneous**	15 (21.4)	15 (21.4)	8 (16)	6(12)	5 (7.1)	6 (8.6)	2 (4)	7 (14)
**Clustered ring**	**0 (0)**	**0 (0)**	**1 (2)**	**1 (2)**	**0 (0)**	**0 (0)**	**3 (6)**	**0 (0)**
**Clumped**	**0 (0)**	**1 (1.4)**	**0 (0)**	**0 (0)**	**0 (0)**	**0 (0)**	**1 (2)**	**1 (2)**
**Microcalcification**								
**Benign microcalcification**	0 (0)	0 (0)	6 (12)	4(8)				
**Suspicious microcalcification**	10 (14.2)	11 (15.7)	4 (8)	7 (14)				
**Microcalcification pattern**								
**Group**	2 (2.8)	0 (0)	3 (6)	1 (2)				
**Segmental**	3 (4.3)	3 (4.3)	0 (0)	5 (10)				
**Regional**	5 (7.1)	3 (4.3)	5 (10)	3 (6)				
**Diffuse**	0 (0)	5 (7.1)	2 (4)	2 (4)				
**Asymmetry**								
**Focal**	1 (1.4)	2 (2.8)	5 (10)	2 (4)				

CEM, contrast-enhanced mammography; MRI, magnetic resonance imaging; Obs, observer.

**Table 4:** Malignant lesion characteristics.

Malignant lesion characteristics - in CEM			- in MRI	
	Obs 1	Obs 2	Obs 1	Obs 2
**Lesion type**	Malignant lesion n (%)		Malignant lesion n (%)	
**Mass enhancement**	51 (72.9)	49 (70)	61 (87)	61 (87)
**Nonmass like enhancement**	5 (7.1)	8 (11.4)	7 (10)	6 (8.6)
**Mass + nonmass-like enhancement**	3 (4.4)	0	0	0
**Nonmass like enhancement + microcalcification**	8 (11.4)	8 (11.4)	0	0
**Mass enhancement + Microcalcification**	1 (1.4)	2 (2.9)	0	0
**Nonenhancement mass**	0	0	2 (3)	3 (4.4)
**Nonenhancement microcalcification**	1 (1.4)	1 (1.4)	0	0
**Nonenhancement Focal asymmetry**	1 (1.4)	2 (2.9)	0	0
**Total**	**70 (100)**		**70 (100)**	

CEM, contrast-enhanced mammography; MRI, magnetic resonance imaging; Obs, observer.
